# Perspective of healthcare providers on assessing the quality and accessibility of health services for chronic diseases in Jordan during Covid-19: a mixed method study

**DOI:** 10.1186/s12913-023-09919-1

**Published:** 2023-08-23

**Authors:** Raya T. Al-Bataineh, Mohammed M. Al-Hammouri, Wafa’a K. Al-Jaraideh

**Affiliations:** 1 Department of Health Management and Policy, College of Medicine, Jordan University of Science and Technology, Irbid, Jordan; 2 Department of Community and Mental Health, College of Nursing, Jordan University of Science and Technology, Irbid, Jordan

**Keywords:** COVID-19, Accessibility, Quality, Chronic diseases, Sociodemographic variables

## Abstract

**Background:**

Hospital services in all parts of the world were severely affected by the crisis caused by the Coronavirus pandemic. This was particularly concerning for patients who suffer from chronic diseases. Aim: This study aimed to: assess the level of quality and accessibility of chronic disease services from the perspectives of healthcare providers, assess the association between healthcare providers’ socio-demographic factors and their perspectives on accessibility and quality level, and explore the providers’ perspectives on the barriers and facilitators of quality and accessibility to chronic disease health services during the COVID -19 pandemic.

**Method:**

Design: An explanatory mixed method design was employed in this study using a questionnaire and focus group discussion approach. The questionnaire consisted of three sections including, demographic, accessibility, and quality. Sample: A convenience sampling approach was used to collect the quantitative from 412 healthcare providers working at public, private, and teaching hospitals. A purposive sample of 12 healthcare providers were interviewed to collect the qualitative data. Analysis: The quantitative data were analyzed using SPSS Statistics Version 25. The qualitative data was analyzed using the thematic analysis approach.

**Results:**

This study found that the quality and accessibility of chronic disease services in northern Jordan were affected during COVID-19. Quantitative: The majority of the participants reported moderate level of accessibility and quality. Qualitative: Four main and six subthemes were identified: 1) Accessibility barriers including transportation and fear of infection; 2) Accessibility facilitators including availability of Personal Protective Equipment (PPE) and Covid-19 vaccination; 3) Quality barriers including staff shortage; 4) Quality facilitators including safety protocol.

**Conclusion:**

The quality and accessibility of chronic disease services were affected due to the healthcare system restating to address the Covid-19 pandemic. Different barriers and facilitators for chronic disease healthcare services accessibility and quality were identified. The findings of this study lay the ground for healthcare decision and policymakers to develop strategies and formulate polices to ensure these patients receive the needed healthcare services, and hence improve their health outcomes.

**Supplementary Information:**

The online version contains supplementary material available at 10.1186/s12913-023-09919-1.

## Introduction

Chronic diseases, also known as no communicable diseases, refers to conditions that last for one year or more, such as but not limited to asthma, cardiovascular diseases, diabetes Miletus (DM), and cancer [1]. Chronic diseases caused by modifiable and non-modifiable factors [2]. Modifiable factors refer to the factors that can be changed and prevented with altered behavior, such as smoking, elevated blood sugar, elevated blood pressure, and obesity [3]. Unmodifiable factors defined as factors that are out of control and cannot be changed, such as gender, race, age, and positive family history [4].

In Jordan, chronic diseases including Diabetes Miletus (DM), cancer, cardiovascular diseases, and chronic respiratory diseases are leading cause of morbidity and mortality. They are responsible for almost 80% of total deaths, with 15% of these deaths occurring in the early thirties and older [5]. Patients with chronic diseases require ongoing medical attention; they should have continuous and timely care to maintain their health status and reduce mortality rates [6, 7]. Patients with unmet healthcare needs have an increased risk of mortality [6, 8].

Unmet healthcare needs due to delay and avoidance of healthcare services became more pronounced during the COVID-19 era [9, 10]. COVID-19, which emerged in China in late 2019, caused world governments to impose a range of mitigating policies, such as social distancing, lockdowns, and curfews, to overcome its impacts [11]. Moreover, healthcare facilities made a substantial shift of financial, technical, and healthcare forces to tackle the high numbers of cases of highly infectious and critically ill patients with COVID-19 [12, 13]. This inevitably caused a major disruption to non-COVID-19 services, including chronic disease services [13–15].

Disruptions of chronic disease services were examined during a rapid assessment conducted by WHO in January 2021, which revealed a disruption of one or more essential chronic disease services among the surveyed countries [16]. The policies put in place to mitigate the spread of the virus resulted in high barriers to accessing healthcare services and a discontinuation of some health services [17]. In addition to accessibility problems, COVID 19 disrupted the primary care system which negatively impacted the quality of healthcare services for patients with chronic diseases [18].

Patients with chronic disease have been shown to suffer from severe symptoms when getting COVID-19 virus [19, 20] with higher mortality rates [21]. In addition to higher susceptibility to infection, these patients are more prone to exacerbation of their condition [22]. Thus, assessing chronic diseases healthcare services quality and accessibility levels during COVID-19 and understanding the factors, including barriers and facilitators, influencing these services is crucial. It could contribute to decreasing the burden of chronic diseases on health and economic systems. Despite the importance of such researches, a lack of studies exists. To our best knowledge, there are no studies that have assessed the quality and accessibility of healthcare services among patients with chronic diseases during COVID-19 in Jordan from the perspectives of healthcare providers. Therefore, this study will help address the gap in the literature regarding healthcare services quality and accessibility among patients with chronic diseases and add to the existing literature on healthcare services in the Middle East. Practically, the study results would give a broader view of the reality of healthcare services in Jordan. Shedding light on the facilitators and barriers of provision of healthcare would lay the ground for developing strategies to improve and sustain healthcare quality and accessibility among patients with chronic disease.

### Aims

The current study has three aims. First, assess level of accessibility and quality of chronic disease services during Covid-19 from the healthcare providers’ perspectives. Second, assess the association between healthcare providers’ socio-demographic factors and their perspectives on accessibility and quality of chronic disease services. Lastly, explore the healthcare providers’ perspectives on barriers and facilitators of accessibility and quality of chronic disease health services during the COVID -19 pandemic.

## Methods

### Design

A sequential explanatory mixed-method design was applied in the study involved the procedure of first gathering quantitative data and then qualitative data. In the quantitative part, a descriptive cross-sectional design was employed as the data was collected using a questionnaire at a single point of time. A focus group discussion approach with semi-structured interviews was used in the qualitative part. Mixed method design was chosen to give a holistic understanding of the phenomena of study. The quantitative part of the study provided information regarding the participating providers’ perspectives on the level of accessibility and quality of healthcare services for patients with chronic diseases. The results revealed that the majority of the participants reported moderate levels of accessibility and quality. Thus, these results informed the second part, a qualitative investigating, that was needed to get a deeper understanding on the reasons/factors impacted the level of accessibility and quality from the healthcare providers’ perspectives.

### Setting

The study was multisite as it was conducted at five main hospitals, including public, teaching, and private hospitals, located in northern Jordan.

### Sample

A total of 412 healthcare providers were recruited using the convenience sampling method. The sample size was determined based on the sample-to-variable ratio, which suggests an observation-to-variable ratio of 15:1 or 20:1 [23]. Using this method, the maximum sample size would be 220 as 11 independent variables (demographic variables) were examined in the study. To better represent the population, larger sample was recruited as the data collected from 412 participants. The participants were selected based on the following inclusion criteria: First, having a direct contact with chronic diseases patients such as physicians, nurses, and pharmacists. Second, having an academic qualification such as diploma, bachelor’s degree, or Master’s.

For the semi-structured interviews, a total of 12 healthcare providers were recruited using the purposive sampling method to acquire in-depth information according to their perspectives [24]. The sample size was determined based on saturation of data.

### Data collection procedure

Before data collection began, the study aims, ethical considerations, inclusion criteria, and the possibility of withdrawing at any point during the research phase were explained to all prospective participants. Those individuals who agreed to participate were asked to provide permission for the researcher to use findings for scientific research and to sign the consent form.

For the quantitative part, the data collection took place in 2021 (September—November) after receiving approval from the Institutional Review Board (IRB) from the Jordan University of Science and Technology and the Jordanian Ministry of Health. The data was collected using a self-reporting questionnaire. Participants who agreed to participate in the study received the study questionnaire to be filled out in a private room at the hospital. The questionnaire was in Arabic language and consisted of three sections: Section one asked about 11 demographic variables including age, gender, marital status, job title, educational level, place of working, place of residence, region, work experience, attending training and development courses, ways of commuting to the workplace. Section two assessed accessibility of chronic disease services using the measure of access (Access-31), which was developed using a literature review and the qualitative method [25]. The modified Access-31 comprised 22 items grouped into five domains including organizational access, geographical access, access to information, cultural acceptability, and availability of services and medicine. Items were rated on a binary scale of no /yes. “No” indicated there was no problems/barrier to accessibility while “Yes” indicated there was a problem/barrier to accessibility. Total scores range between 0 and 22, with a higher score indicating higher level of barriers to accessibility to healthcare services. Judgment was based on cut-off points on scale recommendation. A mean score of zero indicated no barriers, a mean score ranged between (1–3) indicated low level of barriers, a mean score ranged between (4–6) indicated moderate level of barriers, and finally (> 6) indicated high level of barriers toward accessibility to healthcare services. The original measure was modified to be applicable to the study participants. A pilot study of 30 participants was carried out, then reliability was tested using Kuder Richardson. The resulting scores of KR20 = 0.77 and KR20 > 0.7 were considered acceptable. The third section assessed the quality of health care services using a questionnaire developed by Albalasi [26]. The questionnaire consisted of 34 items grouped into three dimensions including responsiveness, assurance, reliability. Items were rated on a 5-point Likert -type scale, ranging from 1 (Strongly disagree) to 5 (Strongly agree). Total scores ranged between 34 and 170, with higher scores indicating better quality of care. The mean score ranged between (1—2.33) indicated low quality, the mean score ranged between (2.34—3.67) indicated moderate quality, and finally the mean score ranged between (3.68—5.0) indicated a high quality of healthcare services. The measure displayed high reliability with a Cronbach alpha of 0.92 [26].

For the qualitative part, the data collection took place in 2022 (June—July). Semi-structured questions were designed to conduct focus group discussion. Focus group approach was used to get in-depth understanding of the participants’ perspective on the study phenomenon [27]. The interviews were conducted in Arabic language to ensure that participants could communicate effectively with the interviewer and express their perspectives more easily.

The total of the twelve healthcare providers were separated into two groups (*n* = 6 for each) based on their availability. The participants were interviewed by the third author, who hold a BSc degree in nursing and MSc degree in healthcare management and quality. The interviews were face-to-face and each interview session lasting approximately 45—60 min. After taking permission from the participants, all discussions were recorded and later only themes and chosen verbatim were translated into English.

### Data analysis

Completed questionnaires were analyzed using descriptive and multiple regression test using IBM SPSS statistics version 25. The sample and study variables were described by measures of central tendency and dispersion appropriate to the level of measurement. The frequencies and percentages are used to represent categorical data such as marital status. Multiple linear regression was used to predict the quality and accessibility of healthcare services using sociodemographic factors. Before conducting multiple regression, Spearman rho, point biserial, and point multi-serial correlation were used as a preliminary step to assess the potential correlations between the independent variables and the outcomes.

The qualitative interview data was analyzed using thematic analysis as described by Kinger et al [28]. Initially, the research team listened to the recorded interviews to get initial interpretation and descriptions of what was saying. The data analysis process continued, as the research team read the transcripts and listened to the recorded interviews several times to fully capture the participant’s words. Line-by-line coding for all the transcripts were conducted and important statements were underlined and extracted from the transcripts. A possible label for the meanings of each statement was formulated. Meanings of the statements then were organized into themes. Passages that have similar themes extracted and compared. Similar themes were grouped and sub-divided till the main themes including the sub-themes were emerged and identified. Lastly, a web meeting was conducted using Microsoft Teams with the participants to discuss and verify the emerged themes. A web meeting was preferred by the participants due to its convenience.

### Results/quantitative

#### Sample characteristics

A total of 412 healthcare providers were enrolled in this study with the majority of the sample was female (60.4%), married (64.1%), nurses (79.9%), working at governmental hospitals (56.3%), holding a bachelor’s degree (70.4%), living in governorate ( 63.6%), and the vast majority were from the north region of Jordan (94.2%). The healthcare providers’ work experience was equally distributed between less than 5 years (39.3%) and 6–15 years group (39.8%). Approximately, (67%) of them were in the age group (20–35 years). The results revealed that more than half of the study sample had attended training and development healthcare courses (63.3%) and used their cars to reach the workplace (59.7%). The detailed characteristics of the study sample are summarized in Table 1.

**Table 1:** Socio-demographic characteristics of study participants (*N* = 412)

**Demographic Variables**	**N (%)**
**Gender**	
- Male	163 (39.6)
- Female	249 (60.4)
**Job title**	
- Nurse	329 (79.9)
- Physician	43 (10.4)
- Pharmacist	40 (9.7)
**Place of work**	
- Governmental Hospital	232 (56.3)
- Private Hospital	100 (24.3)
- Teaching Hospital	80 (19.4)
**Marital status**	
- Married	264 (64.1)
- Unmarried	148 (35.9)
**Educational level**	
- Diploma Degree	57 (13.8)
- Bachelor’s degree	290 (70.4)
- High Degrees	65 (15.8)
**Place of residence**	
- Governorate	262 (63.6)
- Village	150 (36.4)
**Region**	
- North Region	388 (94.2)
- Middle Region	24 (5.8)
**Work experience**	
- Less than 5 years	162 (39.3)
- 6–15 years	164 (39.8)
- Above 16 years	86 (20.9)
**Age**	
- 20–35 years old	275 (66.7)
- 36–51 years old	137 (33.3)
**Attending training and development courses**	
- Yes	261 (63.3)
- No	151 (36.7)
**Way of commuting to the workplace**	
- By car	246 (59.7)
- By public transportation	148 (35.9)
- On foot	18 (4.4)

#### Accessibility of chronic diseases services

Table 2 shows the descriptive statistics for the accessibility measure. The results revealed that the mean of the total score was 4.77(SD 2.80), indicating that, from the perspective of healthcare providers, the chronic disease patients have a moderate level of barriers to healthcare services accessibility according to healthcare providers' perspective. As shown in Table 2, a percentage of (4.6%) of the participants reported no barriers, (31.1%) reported low barriers, (39.5%) reported moderate barriers, and (24.8%) reported high barriers level to chronic disease patients services.

**Table 2:** Barriers level to accessibility to chronic disease patient services from healthcare providers’ perspectives

	Mean ± SD	No Barriers	Low Barriers	Moderate Barriers	High Barriers
Barriers Level to Accessibility to Healthcare Services	4.77 ± 2.8	19 (4.6%)	128 (31.1%)	163 (39.5%)	102 (24.8%)

#### The association between socio-demographic factors and providers’ perspectives on accessibility to chronic disease services

A preliminary bivariate correlation was conducted between healthcare providers' socio-demographic variables and accessibility barriers level to healthcare services. The results of the bivariate correlation test shown in Table 3 revealed that age, place of residence, attending healthcare training and development courses, and work experience were significantly correlated with total score of accessibility barriers level. The sociodemographic variables that were significantly correlated with the level of accessibility barriers were entered later into the regression model.

**Table 3:** Correlation coefficients between healthcare providers' socio-demographic variables and their perspectives on accessibility barriers level to healthcare services

**Variables**	**The total score of accessibility barriers to healthcare services**	
Age	**Point biserial correlation (r**_**pb**_**)**	***P*** ** value**
	-0.130	**0.008**
Place of residence	0.149	**0.002**
Attendance of training and development courses	0.133	**0.007**
Region	0.068	0.169
Marital status	-0.037	0.457
Gender	0.023	0.637
	**Spearman rho correlation (rs)**	***P*** ** values**
Work experience	-0.162	**0.001**
Educational level	0.043	0.386
	**Point Multi-serial correlation (rpm)**	***P*** ** values**
Job title	0.076	0.121
Place of work	0.078	0.114
Way of commuting	0.098	0.109

The result of multiple linear regression in Table 4 demonstrated that the model explained around 6.0% of the variance in the dependent variable and that model was statistically significant (F (5,411) = 6.034, *p* ≤ 0.001), revealing that there is at least one predictor that has a prediction power to predict the score of the dependent variable. The place of residence was a significant positive predictor for accessibility barriers level to healthcare services (B = 0.838, t = 2.994, *p* = 0.003), revealing that living in a village corresponded with increased accessibility barriers level by 0.838 units compared to living in the governorate. Similarly, attending healthcare courses was a significant positive predictor for accessibility to healthcare services (B = 0.763, t = 2.723, *p* = 0.007), demonstrating that not attending training and development healthcare courses corresponded with increased accessibility barriers level to healthcare services by 0.763 units compared to attending training and development healthcare courses. Work experience and age were not statistically significant predictors for accessibility to healthcare services (*P* > 0.05).

**Table 4:** Multiple linear regression of predicting providers’ perspectives on accessibility barriers level to healthcare services

**Dependent Variable**	**Coefficient**						
	**Predictor**	**B**	**SEM**	**T Value**	**Sig**	**VIF**	**Tolerance**
**Accessibility barriers to healthcare services**	Place of residence	0.838	0.28	2.994	**0.003**	1.0	0.99
	Attendance of training and development courses	0.763	0.28	2.723	**0.007**	1.0	0.99
	Age	-0.135	0.41	-0.329	0.742	2.1	0.48
	Experience 1–5 years	0.418	0.33	1.278	0.202	1.4	0.71
	Experience More than 15 years	-0.256	0.15	-1.721	0.086	1.8	0.55

*F* value = 6.034, *R* ^2adj^ = 0.058, *p* ≤ 0.001 Durbin Watson 1.637

#### Quality of chronic diseases services

Table 5 shows the descriptive statistics for the quality measure revealed that the mean score was 3.36 (SD 0.77), indicating that the majority of the healthcare providers in the study reported a moderate level of quality of healthcare services. As shown in Table 5, only (10.2%) of the providers reported a low quality level, half of the sample ( 53.4%) reported a moderate quality level, and (36.4%) of the participants reported high-quality level.

**Table 5:** Healthcare provider's perspectives on quality level of healthcare services

	Mean ± SD	Low Quality	Moderate Quality	High Quality
Quality of Healthcare Services	3.36 ± 0.77	42 (10.2%)	220 (53.4%)	150 (36.4%)

#### Association between socio-demographic factors and providers’ perspectives on quality level of chronic disease services

A preliminary bivariate correlation was conducted between healthcare providers’ socio-demographic variables and quality of healthcare services mean score to explore the significantly correlated variables to enter them later into the regression model. The results shown in Table 6 revealed that age, place of residence, attending training and development healthcare courses, educational level, and work experience were significantly correlated with mean score of the healthcare services quality level.

**Table 6:** Correlation coefficients between healthcare providers' socio-demographic variables and their perspectives on quality of healthcare services

**Variables**	**The total score of quality of healthcare services**	
Age	**Point biserial correlation (r**_**pb**_ **)**	***P*** ** value**
	0.129	**0.009**
Place of residence	-0.097	**0.048**
Attendance of training and development courses	-0.112	**0.022**
Region	-0.079	0.108
Marital status	0.047	0.340
Gender	0.016	0.751
	**Spearman rho correlation (rs)**	***P*** ** values**
Work experience	0.120	**0.015**
Educational level	-0.123	**0.016**
	**Point Multi-serial correlation (rpm)**	***P*** ** values**
Job title	-0.092	0.061
Place of work	-0.056	0.259
Way of commuting	0.053	0.280

The results of multiple linear regression in Table 7 demonstrate that the model explained around 4.0% of the variance in the dependent variable and that model was statistically significant (F (7,411) = 3.455, *p* ≤ 0.001), revealing that there is at least one predictor that has a prediction power to predict the score of the dependent variable. The place of residence was a significant inverse predictor for the quality level of healthcare services (B = -0.159, t = 2.580, *p* = 0.040) revealing that living in a village corresponded with a decrease in the quality level of healthcare services by 0. 159 unit, compared to not living in the governorate. Similarly, not attending training and development healthcare courses was a significant inverse predictor for quality level of healthcare services (B = -0.189, t = 2.437, *p *= 0.015), demonstrating that not attending training and development healthcare courses corresponded with a decrease in the quality level of healthcare services score by 0.189 unit compared to attending training and development healthcare courses. Finally, having a higher degree inversely predicted the quality of healthcare services (B = -0.222, t = -2.139, *p* = 0.033), revealing that providers with higher educational degrees reported lower level of quality of healthcare services. Work experience, diploma degree and age were not significant predictors of quality of healthcare services (*p* > 0.05).

**Table 7:** Multiple linear regression of predicting providers’ perspectives on the quality of healthcare services

**Dependent Variable**	**Coefficient**						
	**Predictor**	**B**	**SEM**	**T Value**	**Sig**	**VIF**	**Tolerance**
**Quality of Healthcare Services**	Place of residence	-0.159	0.077	-2.580	**0.040**	1.0	0.992
	Attendance of training and development courses	-0.189	0.077	-2.437	**0.015**	1.0	0.984
	High degrees	-0.222	0.104	-2.139	**0.033**	1.0	0.962
	Experience 1–5 years	-0.062	0.091	-0.677	0.499	1.4	0.691
	Experience Above 15 years	-0.005	0.041	-0.123	0.903	1.8	0.550
	Diploma degree	0.107	0.111	0.960	0.338	1.1	0.931
	Age	0.188	0.114	1.656	0.099	2.1	0.479

*F* value = 3.455, *R* ^2adj^ = 0.040, *p* ≤ 0.001. Durbin Watson 1.85

#### Results/ qualitative

Twelve healthcare providers were enrolled in this study, with the majority of the sample was female (80%) and living in urban areas (70%). Marital status was distributed equally between married (50%), and single (50%). The detailed characteristics of the study sample are summarized in Table 8.

**Table 8:** Healthcare providers’ demographics *N* = 25: Age (Mean): 43.2 years

Demographics	Frequency
Gender	
Female	80%
Male	20%
Place of residence	
Rural	30%
Urban	70%
Marital status	
Married	50%
Single	50%
Mean age for each group	
Group 1	30.6%
Group 2	28%

The data analysis revealed four major themes and six subthemes, which captured healthcare providers’ perspectives on levels of accessibility and quality of chronic disease health services during COVID-19. As shown in Table 9, two barriers and two facilitators emerged for accessibility, while one barrier and one facilitator emerged for quality.

**Table 9:** Themes and subthemes

**Theme**	**Subthemes**
**1. Accessibility barriers to chronic disease health services during COVID-19, from healthcare providers’ perspectives.**	• Transportation • Fear of infection
**2. Accessibility facilitators to chronic disease health services during COVID-19, from healthcare providers’ perspectives.**	• Availability of Personal Protective Equipment (PPE) • COVID-19 vaccination
**3. Quality barriers to chronic disease health services during COVID-19, from healthcare providers’ perspectives.**	• Healthcare staff shortages
**4. Quality facilitators to chronic disease health services during COVID-19, from healthcare providers’ perspectives.**	• Safety protocols

### Theme 1: accessibility barriers

The participating healthcare providers discussed some barriers to their patients’ accessibility to chronic disease services during COVID-19.

#### Transportation

In our sample, healthcare providers had reported the lack of transportation in their patients’ place of residence relative to the available healthcare facility’s location as a significant barrier to healthcare care accessibility since there is no available transportation to pick them up and take them to healthcare facilities. Participant (1) stated: “I noticed that during appointments, many patients arrived late. I expected to find this issue because of the distance between the patients’ homes and the hospital. Many times, they did not arrive due to the absence or lack of transportation during COVID-19.” Another participant (10) stated: “During COVID-19, I had difficulty to find transportation. For patients, it was harder.”

#### Fear of infection

At the beginning of the pandemic, the knowledge about the symptoms, transmission, and complications of COVID-19 were all vague. This increased the fear among all patients, especially those with chronic diseases due to their lower immunity. This was considered a significant barrier to healthcare services accessibility from the healthcare providers’ perspective. Participant (2) stated: “I know a patient that comes to the hospital routinely to check his blood sugar. During COVID-19, he stopped those visits. I called him by phone and he said, “I will not come to the hospital until the COVID-19 ends. I do not want to get infected.” Another participant (3) stated: “Patients were avoiding their visits to the hospital due to their fear of getting infection if they deal with us. They think that we will transmit the disease to them.”

### Theme 2: accessibility facilitators

The participating healthcare providers discussed some facilitators to their patients’ accessibility to chronic disease services during COVID-19. These include availability of PPE, COVID-19 vaccination.

#### Availability of PPE

During the pandemic, a proactive response of providing PPE (masks, gloves, and gowns) was a facilitator to increasing healthcare services accessibility. Participant (1) stated: “Here in hospital we provide patients with PPE (masks, gloves) for free, and offer services continuously throughout the day, particularly the pharmacy." Another participant (9) stated that: “The presence of gloves and masks has decreased the fear among patients and healthcare providers which increases the accessibility of services.”

#### COVID-19 vaccination

To mitigate the pandemic impacts, the plan for vaccination and increasing immunity was highly adopted. Healthcare service consumers, both patients and healthcare providers, felt less afraid after taking the COVID-19 vaccination. Participant (5) stated: “After the easing of preventive restrictions, chronic disease patients showed a lower degree of fear and they continued their hospital visits because both patients and healthcare providers were vaccinated.” Another participant (1) stated: “After I took two doses of vaccine, I was more comfortable when dealing with patients. In addition, chronic disease patients showed a lower degree of fear after they knew that I was fully vaccinated.”

### Theme 3: quality barriers

The participating healthcare providers discussed one central barrier to chronic disease service quality during COVID-19, namely healthcare staff shortages.

#### Healthcare staff shortages

During COVID-19, a vast number of healthcare staff were infected by the virus. Furthermore, shortages of healthcare staff due to sick leaves were reported as a barrier to quality healthcare services. Participant (3) stated: “My colleagues were infected by COVID-19. There were just two of us at the pharmacy. We can’t advise patients about their medications (insulin) the same as before. I am not satisfied with the service that I delivered, but there is no time due to the workload." Another participant (1) stated: “Workload was higher during COVID-19, where the rest of the staff was infected by the virus. This negatively affected the services that I delivered to patients, especially those with chronic conditions who need counselling”.

### Theme 4: quality facilitators

The participating healthcare providers discussed one central facilitator to chronic disease service quality during COVID-19, namely safety protocols.

#### Safety protocols

During COVID-19, several protocols were enforced to stop disease transmission. The polymerase chain reaction (PCR) test prior to entering the hospital department to ensure individuals were COVID-free before delivering services. Participant (2) stated: “We do not accept COVID-19 patients. Any suspected case was transferred to specialized hospitals. We try to preserve a COVID-19 free area to prevent disease transmission, whether to healthcare staff, to maintain appropriate patient: staff ratio, or for patients with elevated risk.” Participant (5) stated: “The pre-admission PCR test was done to any patient before entering hospital. I was relieved when dealing with those patients where the probability of disease transmission to us is lower.”

## Discussion

### Statement of principal findings

The results of the quantitative phase indicated that healthcare accessibility was impacted by COVID-19. From the healthcare providers’ perspectives, the accessibility level of chronic diseases services was moderate. Additionally, the place of residence and attending healthcare courses were significant positive predictors for providers’ perspective on accessibility to healthcare services. The findings also indicated that the level of the quality of chronic diseases services during covid-19 was moderate. Place of residence, having a higher degree, and not attending training and development healthcare courses were significant inverse predictors for providers’ perspective on quality of healthcare services. Findings of the qualitative phase showed that from healthcare providers’ perspectives, transportation and fear of infection were the most important barriers to access chronic disease services during COVID-19. On the other hand, the availability of Personal Protective Equipment(PPE) and COVID-19 vaccination were perceived as facilitators to access chronic disease services during COVID-19. Health staff shortage was perceived as a quality barrier whereas safety protocol was perceived as a quality facilitator.

### Interpretation within the context of the wider literature

#### Healthcare services accessibility

Our findings indicated that the majority of the participants (95.4%) perceived barriers, ranging from low to high, to patients' accessibility level to chronic disease health services during COVID-19. Our results are in consistent with a previous study [29, 30], which revealed that the COVID-19 pandemic induced impacts on access and utilization of healthcare. In line with the results of the quantitative part, the findings of the qualitative study also showed that the healthcare providers discussed two barriers to patients' accessibility, including transportation and fear of infection. Due to disruption or even lack of public transportation during COVID-19, patients found it difficult to access healthcare. Our findings are in line with a previous study by Bullen et al. [30], in which healthcare providers indicated lack of transportation as a barrier to accessing chronic disease services. Additionally, the healthcare providers reported that the significant explicit level of fear among chronic disease patients increased delaying, discontinuing, and denying seeking healthcare services. This is consistent with a study by Danhieux et al. [9], who stated that healthcare providers reported a decrease in patients’ visits during COVID-19 due to the fear of catching COVID-19. Besides the barriers to accessibility, the participants reported two facilitators, including the availability of Personal Protective Equipment(PPE) and the COVID-19 vaccination. The participants reported that providing patients with masks, gloves, and other PPE for free decreased the fear among both patients and healthcare providers and hence facilitate access to healthcare services. This could be contributed to the role of PPE in decreasing transmission of COVID-19. This result is consistent to existing evidence [31] on the association between the availability of PPE and reduction in COVID-19 infection rate. The second facilitator perceived by healthcare providers was the COVID-19 vaccination. In our study, the participating healthcare providers revealed that their patients who got vaccinated showed a lower degree of fear toward accessing healthcare services due to the vaccination of both patients and healthcare providers. This is consistent with a previous study [32], which indicated that the vaccination was associated with lower fear of infection.

The results of the multiple regression in the quantitative part of the study revealed that there were two socio-demographic factors including, place of residence and training and development courses attendance significantly correlated with healthcare providers’ perspectives toward accessibility barriers level. Our study revealed that providers live in a village reported higher barriers level toward accessibility to healthcare services compared to living in the governorate. The findings in this study remained consistent with previous research [33] that revealed a significant and strong association between place of residence and accessibility to chronic disease health services. The study [33] found that living in rural areas presents barriers to accessing health services as perceived by healthcare professionals in the study. Another study conducted in the United States [34] revealed that health professionals in rural areas perceive impacts on access to health services during COVID-19. The study results also showed that not attending courses associated with reporting higher accessibility barriers level. To the best of our knowledge, there is no studies investigating the association between healthcare training and perspectives toward healthcare services accessibility. However, access definition is not limited to the physical exist of the service. It also referrers to successful use of the service [35]. Based on that, the current result could be explained that trained providers compared to untrained could communicate with patients and identify and their health needs more effectively, and accordingly enhance their accessibility.

#### Healthcare services quality

The findings from the quantitative part revealed that the level of quality of chronic diseases services was impacted by COVID -19, as revealed by the fact that more than the half (63.6%) of the healthcare providers reported low to moderate quality services during COVID-19. Our findings are in line with a previous study from Belgium, which pointed that chronic diseases service quality was changed during COVID -19 due to the disruption of services [9]. The qualitative results supported the quantitative results as it showed that healthcare staff shortage due to COVID-19 sick leaves was reported as a barrier to quality healthcare services. As reported by the healthcare providers, being overloaded and the extended demand of COVID-19 pandemic negatively influenced the quality of provided services. The study results remained consistent with a previous study [36], in which healthcare worker shortages was perceived as a significant barrier toward delivering quality chronic disease healthcare services. The qualitative part of the study also revealed that safety protocols was a quality facilitator. As reported by the participants, requiring COVID-19 polymerase chain reaction (PCR) test prior to entering the hospital made them more comfortable to work with patients since the probability of disease transmission to them is low. This result is in line with a previous study [31], which revealed that a high satisfaction rate was recorded among healthcare providers due to the application of institutional protocols. They believe that institutional protocols decrease the risk of infection for both patients and healthcare providers.

As revealed by the quantitative part, three sociodemographic factors including, place of residence; educational level; and training and development courses attendance significantly correlated with providers’ perspectives on quality of healthcare services. Our study revealed that living in a village corresponds with a decrease in the participants’ perspectives on quality level of healthcare services compared to those who live in the governorate. This is could be attributed to inefficiencies or maldistribution of resources in rural areas. The result is consistent with a previous study, which indicated that people live in rural areas face several challenges for getting quality care [37]. Additionally, our results revealed that not attending training and development healthcare corresponded with a decrease in the providers’ perspectives on quality of healthcare services score unit compared with those who attending such courses. This could be attributed to the fact that training courses during COVID-19 for health providers served to improve their knowledge of the consequences and prevention methods of COVID-19 pandemic and may reflect on their competency to enhance patients’ outcomes [38]. The current findings remained consistent with previous research that pointed out that healthcare providers who attended training courses have a higher perception of healthcare quality [26]. The study results revealed that providers who had higher educational degrees reported lower level of quality of healthcare services. Patients with higher education expect high standard care [39]. It could be the same for healthcare providers. In other words, providers with higher education achievement have higher medical knowledge and awareness, and accordingly expect high quality care.

## Study limitations

The current study has five limitations: First, the current study used a convenience sampling approach, which may limit the ability to generalize the study findings across the target population [40]. Nevertheless, conducting the study at multiple sites, including public, private, and university hospitals and the potential diversity of geographical and socioeconomic backgrounds among the participants should maximize the external validity of the study, and thus enhance the generalizability of the results. Second, self-reporting measures were used to collect quantitative data, increasing the likelihood of producing response or social desirability bias, and in turn, limiting internal validity [41]. However, the study measures were used previously and have been shown to be reliable and valid measures. Further, the participants were encouraged to respond to questionnaire items truthfully, they were assured that the questionnaire would not be linked to them personally in any way, and they filled out the questionnaire privately. Third, the qualitative data was collected using focus groups. Individual interviews could give interviewers deeper understanding of the participants’ experiences and opinions. However, focus groups allow interviewers to gather a broad range of opinions [42, 43] and allow for productive dialogue among the participants, especially the size of the focus groups in the current study was optimal [44]; it was not too large neither too small. Fourth, the majority of the sample in the qualitative part was female. Five, no military hospitals were included among the study hospitals. Despite these limitations, this study contributes significantly to the field of research on the quality and accessibility of chronic disease health services, filling previously identified research gaps. To the best of the researcher’s knowledge, this is the first study in Jordan assessing the level of both quality and accessibility, exploring barriers and facilitators perceived by healthcare providers, and assessing the prediction effect of sociodemographic on healthcare providers’ perspectives on quality and accessibility. Most existing studies focus on the disruption of services caused by the spread of COVID-19.

## Conclusions

In sum, both of quality and accessibility of healthcare services of patients with chronic diseases have been impacted by the restructuring of healthcare systems to address the COVID-19 pandemic in Jordan. The results of this study are consistent with the literature on the changes in the quality and accessibility of chronic disease services caused by the pandemic. Nevertheless the probability of having a similar pandemic in the future is low, the findings of the study could help policymakers to strengthen their preparedness to any unexpected future crisis taking into consideration the defined health care services facilitators and barriers of these vulnerable population. The findings of the study shed the light on some of the strength and weakness points of the Jordanian national response to Covid-19 that could be learned from. The study also identified different quality and accessibility barriers and facilitators, which impact patients' health status, outcome, and continuation of a treatment plan. Being aware of these factors lay the ground for policy and decision makers to develop strategies and formulate polices to meet the need of these patients, and hence maintain or improve their health outcomes. For example, transportation and staff shortage were revealed as barriers for healthcare accessibility and quality, respectively. Although these issues become more obvious during COVID-19, they have been among the consistent challenges in healthcare sector [45, 46]. Individuals living in rural areas or with lower socioeconomic status face greater challenges to reach healthcare facilities even prior to COVID-19 [45]. Therefore, attention need to be given for increasing availability and accessibility to affordable transportation for these patients. Moreover, the findings stress the importance for healthcare organizations to implement workforce strategies to attract or retain qualified healthcare workers.

Investigating whether the quality and accessibility for these patients remain at the same level or get improved after the pandemic in future studies is recommended. Furthermore, conducting future longitudinal research is recommended to determine whether there are any adverse outcomes for these patients. The most important take-away message, especially from the qualitative part, is that the majority of the participants were aware of the impact of COVID-19 on their patients’ healthcare services accessibility and quality and they strived to do the best for them.

### Supplementary Information

**Additional file 1.**

## Data Availability

The Data will be available upon a reasonable request from corresponding author Raya T. Albataineh by email (rtalbataineh0@just.edu.jo).

## References

[1] Raghupathi W, Raghupathi V (2018). An empirical study of chronic diseases in the United States: a visual analytics approach to public health. Int J Environ Res Public Health.

[2] World Health Organization (WHO). https://www.who.int/news-room/fact-sheets/detail/noncommunicable-diseases. Accessed 10 Oct 2022. [Cited 2021 Jun 7].

[3] Hoy D, Rao C, Nhung NT, Marks G, Hoa NP. Peer Reviewed: Risk Factors for Chronic Disease in Viet Nam: A Review of the Literature. Prev Chronic Dis. 2013;10. 10.5888/pcd10.120067 PMC3545704 23306076

[4] Praus F, Schönthaler M (2019). Modifiable and non-modifiable risk factors for urolithiasis. Urologe.

[5] World Health Organization (WHO). https://www.emro.who.int/jor/jordan-news/results-of-jordan-national-stepwise-survey-steps-of-noncommunicable-diseases-and-their-risk-factors-2019.html. Accessed 19 May 2023.

[6] Herr M, Arvieu JJ, Aegerter P, Robine JM, Ankri J (2014). Unmet health care needs of older people: prevalence and predictors in a French cross-sectional survey. Eur J Public Health.

[7] Locatelli SM, Sharp LK, Syed ST, Bhansari S, Gerber BS (2017). Measuring health-related transportation barriers in urban settings. J Appl Meas.

[8] Di Cesare M, Khang Y-H, Asaria P, Blakely T, Cowan MJ, Farzadfar F (2013). Inequalities in non-communicable diseases and effective responses. Lancet.

[9] Danhieux K, Buffel V, Pairon A, Benkheil A, Remmen R, Wouters E (2020). The impact of COVID-19 on chronic care according to providers: a qualitative study among primary care practices in Belgium. BMC Fam Pract.

[10] Czeisler MÉ, Marynak K, Clarke KE, Salah Z, Shakya I, Thierry JM, Ali N, McMillan H, Wiley JF, Weaver MD, Czeisler CA (2020). Delay or avoidance of medical care because of COVID-19–related concerns—United States, June 2020. Morb Mortal Wkly Rep.

[11] Ayouni I, Maatoug J, Dhouib W, Zammit N, Fredj SB, Ghammam R, Ghannem H (2021). Effective public health measures to mitigate the spread of COVID-19: a systematic review. BMC Public Health.

[12] The U.S. Department of Health and Human Services. Impact of the COVID-19 Pandemic on the Hospital and Outpatient Clinician Workforce. Available online: https://aspe.hhs.gov/sites/default/files/documents/9cc72124abd9ea25d58a22c7692dccb6/aspe-covid-workforce-report.pdf. Accessed 10 Sept 2022.

[13] Luciani S, Caixeta R, Chavez C, Ondarsuhu D, Hennis A (2023). What is the NCD service capacity and disruptions due to COVID-19? Results from the WHO non-communicable disease country capacity survey in the Americas region. BMJ Open.

[14] Allameh SF, Amiri BS, Jalalabadi NZ (2020). Disruption in medical care of non-COVID patients in COVID-19 pandemic. Front Emerg Med.

[15] World Health Organization (2020). The impact of the COVID-19 pandemic on noncommunicable disease resources and services: results of a rapid assessment.

[16] World Health Organization (WHO). https://www.who.int/publications/i/item/WHO-2019-nCoV-EHS-continuity-survey-2021. [Cited 2021 October 7].

[17] Nshimyiryo A, Barnhart DA, Cubaka VK, Dusengimana JMV, Dusabeyezu S, Ndagijimana D (2021). Barriers and coping mechanisms to accessing healthcare during the COVID-19 lockdown: a cross-sectional survey among patients with chronic diseases in rural Rwanda. BMC Public Health.

[18] Basu S (2020). Non-communicable disease management in vulnerable patients during Covid-19. Indian J Med Ethics.

[19] ADA (2022). Diabetes and Coronavirus (COVID-19): How COVID-19 Impacts People with Diabetes. American Diabetes Association. https://diabetes.org/coronavirus-covid-19/how-coronavirus-impacts-people-with-diabetes.

[20] Liu N, Huang R, Baldacchino T, Sud A, Sud K, Khadra M, Kim J (2020). Telehealth for noncritical patients with chronic diseases during the COVID-19 pandemic. J Med Internet Res.

[21] Singh AK, Misra A (2020). Impact of COVID-19 and comorbidities on health and economics: focus on developing countries and India. Diabetes Metab Syndr.

[22] Sawaya T, Ballouz T, Zaraket H, Rizk N (2020). Coronavirus disease (COVID-19) in the Middle East: a call for a unified response. Front Public Health.

[23] Memon MA, Ting H, Cheah J-H, Thurasamy R, Chuah F, Cham TH (2020). Sample size for survey research: review and recommendations. J Appl Struct Equ Model.

[24] Palinkas LA, Horwitz SM, Green CA, Wisdom JP, Duan N, Hoagwood K (2015). Purposeful sampling for qualitative data collection and analysis in mixed method implementation research. Adm Policy Ment Health.

[25] Alasfoor D. Exploring access to primary health care among diabetic patients. Oman: University of Oxford; 2020.

[26] Balasi A. Assessing the quality of services at UNRWA Health Care Centers at Hebron and Bethlehem Areas from patients and professionals point of views. Int Humanit Stud. 2016;3(4):50–52.

[27] Krueger RA. Focus groups: A practical guide for applied research. Thousand Oaks: Sage publications; 2014.

[28] Kiger ME, Varpio L (2020). Thematic analysis of qualitative data: AMEE Guide No. 131. Med Teach.

[29] Chudasama YV, Gillies CL, Zaccardi F, Coles B, Davies MJ, Seidu S, Khunti K (2020). Impact of COVID-19 on routine care for chronic diseases: a global survey of views from healthcare professionals. Diabetes Metab Syndr.

[30] Bullen C, McCormack J, Calder A, Parag V, Subramaniam K, Majumdar A (2021). The impact of COVID-19 on the care of people living with noncommunicable diseases in low-and middle-income countries: an online survey of physicians and pharmacists in nine countries. Prim Health Care Res Dev..

[31] Nemat A, Alsarhan O, Raufi N, Al Zein EI, Kheirallah KA, Mubarak MY (2021). Availability of personal protective equipment among health-care workers in Jordan during the COVID-19 pandemic: a web-based survey. Risk Manag Healthc Policy.

[32] Hatmal MM, Al-Hatamleh MA, Olaimat AN, Hatmal M, Alhaj-Qasem DM, Olaimat TM, Mohamud R (2021). Side effects and perceptions following COVID-19 vaccination in Jordan: a randomized, cross-sectional study implementing machine learning for predicting severity of side effects. Vaccines.

[33] Hoerold M, Gottschalk M, Debbeler CM, Heytens H, Ehrentreich S, Braun-Dullaeus RC (2021). Healthcare professionals’ perceptions of impacts of the Covid-19-pandemic on outpatient care in rural areas: a qualitative study. BMC Health Serv Res.

[34] Coombs NC, Campbell DG, Caringi J (2022). A qualitative study of rural healthcare providers’ views of social, cultural, and programmatic barriers to healthcare access. BMC Health Serv Res.

[35] Alborz A, McNally R, Glendinning C (2005). Access to health care for people with learning disabilities in the UK: mapping the issues and reviewing the evidence. J Health Serv Res Policy.

[36] Kamvura TT, Dambi JM, Chiriseri E, Turner J, Verhey R, Chibanda D (2022). Barriers to the provision of non-communicable disease care in Zimbabwe: a qualitative study of primary health care nurses. BMC Nurs.

[37] Moscovice I, Rosenblatt R (2000). Quality-of-care challenges for rural health. J Rural Health.

[38] Hussain I, Majeed A, Imran I, Ullah M, Hashmi FK, Saeed H, Chaudhry MO, Rasool MF (2021). Knowledge, attitude, and practices toward COVID-19 in primary healthcare providers: a cross-sectional study from three tertiary care hospitals of Peshawar, Pakistan. J Community Health.

[39] Dikmen Y, Yılmaz D (2016). Patient’s perceptions of nursing care-a descriptive study from Turkey. Ann Nurs Pract.

[40] Andrade C (2021). The inconvenient truth about convenience and purposive samples. Indian J Psychol Med.

[41] Althubaiti A (2016). Information bias in health research: definition, pitfalls, and adjustment methods. J Multidiscip Healthc.

[42] O. Nyumba T, Wilson K, Derrick CJ, Mukherjee N (2018). The use of focus group discussion methodology: Insights from two decades of application in conservation. Methods Ecol Evol.

[43] Bourgeault I, de Vries R, Dingwall R. The SAGE handbook of qualitative methods in health research. The SAGE Handbook of Qualitative Methods in Health Research. 2010. p. 1–786.

[44] Stalmeijer RE, McNaughton N, Van Mook WN (2014). Using focus groups in medical education research: AMEE Guide No. 91. Med Teach.

[45] Arcury TA, Preisser JS, Gesler WM, Powers JM (2005). Access to transportation and health care utilization in a rural region. J Rural Health.

[46] Winter V, Schreyögg J, Thiel A (2020). Hospital staff shortages: environmental and organizational determinants and implications for patient satisfaction. Health Policy.

