# Supplementary material for: Perspective of healthcare providers on assessing the quality and accessibility of health services for chronic diseases in Jordan during Covid-19: a mixed method study
Source: BMC Health Serv Res. 2023 Aug 23;23:895. doi: 10.1186/s12913-023-09919-1 (PMC10464245; doi:10.1186/s12913-023-09919-1)
Supplement: Supplementary file 1 — Additional file 1. [file 12913_2023_9919_MOESM1_ESM.docx]

**Supplementary Material**

**Study instrument (Healthcare Provider) / Arabic Copy**


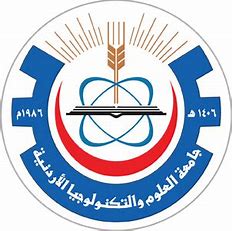


نموذج دعوة مشاركة

استبانة مقدمي الرعاية الصحية

**أنا الطالبه " وفاء خالد ارشيد" من تخصص ادارة الرعاية الصحية و الجودة - كلية الطب - أدعوكم للمشاركة في الدراسة التي تهدف الى** قياس اثر جائحة كورونا على جودة وسهولة الوصول للخدمات الصحية المقدمة لأصحاب الامراض المزمنة في الاردن من ( وجهة نظر المرضى ومقدمي الرعاية الصحية**)**

**الرجاء التكرم بالاجابة على الاسئلة المرفقة بدقة وموضوعية ، علما ان كافة الاجابات ستعامل بسرية تامة وستستخدم لاغراض البحث العلمي فقط ، وأؤكد انه لا وجود لأي أسئلة تشير الى هوية المجيب .**

شاكرين مساعدتكم وحسن تعاملكم

تقييم جودة وسهولة الوصول للخدمات الصحية المقدمة لأصحاب الامراض المزمنة خلال جائحة كورونا في الاردن من ( وجهة نظر المرضى ومقدمي الرعاية الصحية)

**القسم الاول: معلومات عامة**

1- العمر :20-35 🔾 36-51 🔾

2- الجنس: ذكر 🔾 انثى 🔾

3- الحالة الاجتماعية: غير متزوج 🔾 متزوج 🔾

4- المؤهل العلمي: دبلوم 🔾 بكالوريوس 🔾 دراسات عليا 🔾

5- مكان السكن: محافظة 🔾 قرية 🔾

6- الاقليم: شمال 🔾 غير ذلك 🔾

7- المسمى الوظيفي: ممرض 🔾 طبيب 🔾 صيدلي 🔾

8- نوع الخدمة الصحية: خاص 🔾 حكومي 🔾 تعليمي 🔾

9- عدد سنوات الخبرة: اقل او يساوي 5 سنوات 🔾 6-15🔾 اكثر من 16🔾

10- هل حضرت دورات او محاضرات في مجال الخدمة الصحية التي تقدمها خلال العام الماضي ؟.

نعم 🔾 لا 🔾

11- كيف تذهب إلى مكان عملك ؟

سياره خاصه 🔾 مواصلات عامه 🔾 مشيا على الاقدام 🔾

**القسم الثاني :استبيان سهولة الوصول للخدمات الصحية**

12- كم من الوقت يستغرقك عادة للوصول الى العمل؟ اقل من نصف ساعه 🔾 نصف ساعه فاكثر 🔾

13- في خلال ١٢ شهرا الماضية هل حصل لك وتخلفت أو تأخرت عن موعد العمل بسبب عدم وجود مواصلات؟

نعم 🔾 لا 🔾

14- في خلال ١٢ شهرا الماضية هل حصل لك وتخلفت أو تأخرت عن موعد العمل بسبب عدم قدرتك على دفع سعر المواصلات؟

نعم 🔾 لا 🔾

15- هل يقوم الطبيب أو المركز الصحي /المستشفى بالاتصال بالمرضى لإبلاغهم عن نتائج الفحوص المختبرية؟

نعم 🔾 لا 🔾

16- هل يتم توفير المعلومات للمرضى حول المحافظة على صحتهم ؟

نعم 🔾 لا 🔾

17- هل تقدم النصائح حول ما يقلق المريض من الناحية الصحية؟

نعم 🔾 لا 🔾

18- إذا تلقيت اتصال بالمركزالصحي /المستشفى  للاستفسار حول اسئلة خلال ساعات العمل الرسمية هل تقدم اجابة في نفس اليوم؟

نعم 🔾 لا 🔾

19- إذا تلقيت اتصال بالمركز الصحي/المستشفى للاستفسار حول أسئلة بعد ساعات العمل الرسمية هل تقدم إجابة في نفس اليوم؟

نعم 🔾 لا 🔾

20- هل يعرف المرضى كيفية الحصول على مواعيد في المساء وأوقات الاجازات؟

نعم 🔾 لا 🔾

21- كم الوقت المقدر للانتظار منذ وصول المريض وحتى مقابلة الطبيب؟

اقل او يساوي ساعة 🔾 اكثر من ساعة 🔾

22- كم من الوقت ينتظر المريض لمقابلة الاخصائي او الاستشاري ؟

اقل او يساوي شهر 🔾 اكثر من شهر 🔾

23- إذا كان المريض غير قادر على الوصول للمركز الصحي/المستشفى بسبب إصابة أو إعاقة، هل يعرف كيف يتواصل مع الطبيب للحصول على الرعاية اللازمة؟

نعم 🔾 لا 🔾 لا ينطبق 🔾

24- هل تتحدث مع المريض بلغة يفهمها؟

نعم 🔾 لا 🔾

25- في الماضي ، هل واجهت صعوبة في فهم المريض ؟

نعم 🔾 لا 🔾

26- هل قدمت معلومات مقروءة أو غيرها للمرضى بخصوص مرضهم ؟

نعم 🔾 لا 🔾

27- عندما تناقش حالة المريض الصحية أو ما يقلقه  ، هل يحصل  دائما على مكان به خصوصية ، غرفة خاصة مثلا؟

نعم 🔾 لا 🔾

28- في 12 شهرا الماضية هل حصل أن شهدت او تعاملت بطريقة غير محترمة مع المرضى في المركز الصحي/المستشفى ؟

نعم 🔾 لا 🔾

29- عندما تتحدث مع المريض ، هل تشرح له مرضه وتجيب على اسئلته بأريحية؟

نعم 🔾 لا 🔾

30- في 12 شهرا الماضية هل تكلمت مع المريض حول الأدوية التي يتناولها ، بما فيها تلك التي وصفتها له ؟

نعم 🔾 لا 🔾

31- خلال السنة الماضية ، هل وصفت دواء باعتقادك لن يتمكن المريض من الحصول عليه؟

نعم 🔾 لا 🔾

32- اذا طلبت من المريض إجراء فحص دم او اي فحوصات اخرى هل تتوفر دائما في المركز الصحي/المستشفى أو المجمع الصحي؟

نعم 🔾 لا 🔾 لم اطلب اجراء فحص دم 🔾

33- اذا ما احتاج المريض الى زيارة أخصائي تغذية ، العلاج الطبيعي أو أي مختص آخر هل هم يكونوا متواجدين في المركز الصحي باستمرار ؟

نعم 🔾 لا 🔾

لم اطلب ذلك 🔾

**القسم الثالث: معلومات حول المركز الصحي الذي تعمل به**

نرجو منك قراءة العباارت الآتية بعناية، والإجابة عنها بوضع إشارة( √) في العمود الذي تتفق معه:

| **غير موافق على الاطلاق** | **غير موافق** | **موافق بدرجة متوسطة** | **موافق** | **موافق جدا** | **العبارة** | **الرقم** |
| --- | --- | --- | --- | --- | --- | --- |
|  |  |  |  |  | يتبع هذا المركز الصحي الخطة الإستراتيجية لوزارة الصحة | 34 |
|  |  |  |  |  | تسمح بيئة العمل في المركز الصحي/المستشفى بتطبيق الخطة الإستراتيجية | 35 |
|  |  |  |  |  | يوجد في المركز الصحي/المستشفى بروتوكول لمراجعة السياسات التي تحكم سير العمل | 36 |
|  |  |  |  |  | يوجد في المركز الصحي/المستشفى خطط لإدارة الجودة | 37 |
|  |  |  |  |  | يتم عمل تقييم لجودة الخدمة الصحية في هذا المركز الصحي/المستشفى بشكل سنوي | 38 |
|  |  |  |  |  | تسمح البيئة الإدارية للمركز الصحي/المستشفى بتحسين جودة العمل | 39 |
|  |  |  |  |  | يوجد في المركز الصحي/المستشفى نظام مكتوب لتحفيز الموظفين | 40 |
|  |  |  |  |  | يوجد في المركز الصحي/المستشفى نظام مكتوب لتقييم اداء الموظفين | 41 |
|  |  |  |  |  | يشارك الموظفون في الاجتماعات الإدارية للمركز الصحي/المستشفى | 42 |
|  |  |  |  |  | تأخذ ادارة المركز الصحي/المستشفى باقتراحات و ارء الموظفين | 43 |
|  |  |  |  |  | زادت القرارات الإدارية في هذا المركز/المستشفى من جودة الخدمات الصحية | 44 |
|  |  |  |  |  | أعتقد بأن الرواتب الشهرية والعلاوات منصفة وتلائم طبيعة مهام الموظفين | 45 |
|  |  |  |  |  | لا اشعر بالعزلة في هذا المركز الصحي/المستشفى | 46 |
|  |  |  |  |  | تقوم الدائرة الصحية لوزارة الصحة بالاتصال وزيارة هذا المركز/المستشفى باستمرار | 47 |
|  |  |  |  |  | يتمتع العاملين في المركز الصحي بعلاقات مهنية جيده فيما بينهم | 48 |
|  |  |  |  |  | يتم عمل تقييم العلاقات بين الطاقم الإداري والعاملين بشكل منظم | 49 |
|  |  |  |  |  | تتوزع المهمات الوظيفية بين العاملين في هذا المركز/المستشفى بشكل عادل | 50 |
|  |  |  |  |  | تتوزع المسؤوليات الوظيفية بين العاملين في هذا المركز/المستشفى بشكل عادل | 51 |
|  |  |  |  |  | بيئة العمل في هذا المركز الصحي/المستشفى مريحة | 52 |
|  |  |  |  |  | يوجد علاقة تعاون بين هذا المركز الصحي/المستشفى ومؤسسات المجتمع المحلي | 53 |
|  |  |  |  |  | يتعاون الكادر الصحي في أداء مهامهم كفريق واحد | 54 |
|  |  |  |  |  | اعتقد بان المركز الصحي/المستشفى يستطيع تحقيق كافة حاجات المرضى الصحية | 55 |
|  |  |  |  |  | يتفهم موظفو المركز الصحي /المستشفى احتياجات المرضى الخاصة | 56 |
|  |  |  |  |  | يمتلك العاملون الخبرة المهنية للرد على استفسا ارت المرضى | 57 |
|  |  |  |  |  | تتوفر باستمرار الادوية الضرورية لاحتياجات المرضى | 58 |
|  |  |  |  |  | يتم الاستجابة لطلب المريض إذا كان يرغب في الحصول على مضاد حيوي | 59 |
|  |  |  |  |  | يسمح ضغط العمل بأن يؤدي العاملين واجباتهم بجودة عالية | 60 |
|  |  |  |  |  | يتوفر لدى المركز الصحي/المستشفى معدات وأجهزة حديثة كافيه لتقديم الرعاية الصحية | 61 |
|  |  |  |  |  | تتناسب مرافق وقاعات المركز الصحي الداخلية مع طبيعة الخدمات الصحية المتوفرة | 62 |
|  |  |  |  |  | نظام مواعيد المرضى المتبع في المركز الصحي/المستشفى يسهل على الموظفين اداء واجباتهم بسهولة | 63 |
|  |  |  |  |  | يحتفظ المركز الصحي/المستشفى بسجلات طبية للمرضى دقيقة وموثقة | 64 |
|  |  |  |  |  | عدد افراد الطاقم الصحي كافي ويتناسب مع عدد المرضى والمراجعين | 65 |
|  |  |  |  |  | اشعر بالحاجة إلى تلقي تدريب إضافي في مجال الرعاية الصحية الشاملة | 66 |
|  |  |  |  |  | يحافظ المركز الصحي /المستشفى على استمرار تدريب وتطوير العاملين فيه | 67 |

**Qualitative Study Instrument / Focus Group Discussion Questions**

**
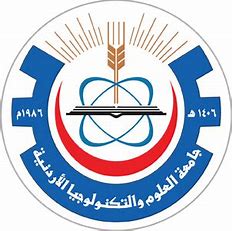
**

**موافقة على الاشتراك في دراسة بحثية**

انت مدعو للمشاركة في هذه الدراسة التي تهدف الى قياس أثر جائحة كورونا على جودة وسهولة الوصول للخدمات الصحية المقدمة لأصحاب الامراض المزمنة في الاردن - وجهة نظر المرضى ومقدمي الرعاية الصحية - وهي جزء لاستكمال رسالة الماجستير في تخصص ادارة الرعاية الصحية والجودة - كلية الطب /جامعة العلوم والتكنولوجيا الاردنية

قبل موافقتك على المشاركة بالدراسة ستقوم الباحثة بتلخيص المعلومات المهمة التي ستسمح لك باتخاذ قرار المشاركة او عدمه، يجوز لك عدم المشاركة في الدراسة اوالانسحاب في اي وقت دون اي عواقب،ستعامل كافة المعلومات بسرية تامة ولن تستخدم الا لاغراض البحث العلمي ،ترغب الباحثة في تسجيل جلسة النقاش لتسهيل تحويل وترجمة المعلومات المحكية الى مكتوبة ،لن تربط المعلومات باسم المشارك وستكون بصيغة مجهول الهوية .

الرجاء الذهاب للصفحة التالية والاجابة عن بعض الاسئلة بدقة وموضوعية ويمكنك السؤال والاستفسار بحرية بما يتعلق بموضوع الدراسة ،راجين منكم الحفاظ على سرية المعلومات والبيانات في هذه الجلسة النقاشية .

**شاكرين مساعدتكم وحسن تعاملكم**

**🔾أوافق**

**🔾 لا أوافق**

**تقييم جودة وسهولة الوصول للخدمات الصحية المقدمة لأصحاب الامراض المزمنة خلال جائحة كورونا في الاردن :-وجهة نظر مقدمي الرعاية الصحية –**

**معلومات عامة :**

- العمر :............
- الجنس: ذكر/انثى
- التدخين: مدخن/ة/غيرمدخن/ة
- الحالة الاجتماعية:متزوج/ة/غير متزوج/ة
- مكان السكن :مدينة/قرية
- هل حضرت دورات او محاضرات في مجال الخدمة الصحية التي خلال العام الماضي ؟ نعم/لا ؟ما مدى رضاك عنها؟

**أسئلة الدراسة :**

1. ما هي المشاكل التي واجهتك لتسهيل وصول المرضى الى الخدمة الصحيه خلال جائحة كورونا ؟
2. اذكر أهم النقاط التي ساعدتك لتسهيل وصول المرضى الى الخدمة الصحية اثناء جائحة كورونا ؟
3. ماهي اهم الاجراءات التي تقترحها لتسهيل وصول المرضى للخدمات الصحية في المستقبل ؟
4. باعتقادك ،هل ساعد لقاح كورونا في تسهيل الوصول للخدمات الصحية خلال الجائحة ؟كيف؟
5. ما هي المشاكل التي واجهتك لتقديم خدمات صحية ذات جودة خلال جائحة كورونا؟
6. اذكر أهم النقاط التي ساعدتك لتقديم خدمات صحية ذات جودة خلال جائحة كورونا؟
